# Anisakiasis and Anisakidae

**DOI:** 10.3390/pathogens13020148

**Published:** 2024-02-06

**Authors:** Francisco Javier Adroher, Manuel Morales-Yuste, Rocío Benítez

**Affiliations:** Department of Parasitology, Faculty of Pharmacy, University of Granada, 18071 Granada, Spain

Parasitism as a lifestyle is much more common in nature than it seems. It is easy to think that the parasites make life difficult for free-living beings. If there were only one parasite species per host, there would be as many parasitic species as free-living ones. However, by observing nature and the relationships between living beings, we observe that many of them have more than one specific parasitic species, so we can easily say that there are more parasitic life species than free-living ones [1], especially when we consider that some parasite species are themselves parasitised, which is known as hyperparasitism. Indeed, parasitism stands as a prominent lifestyle within nature, accounting for a significant proportion of biomass and production within specific ecosystems [2,3]. Hence, there is great interest and importance in studying parasites and their relationships with their hosts. Humans also have numerous parasites that can become temporarily or permanently established, causing mild to severe damage and, occasionally, death.

Anisakidosis or anisakiasis is an underdiagnosed, emerging, cosmopolitan disease. This condition in humans results from the accidental ingestion of the third larval stage (L3) of parasitic nematodes belonging to the family Anisakidae (genera *Anisakis*, *Pseudoterranova*, and, very rarely, *Contracaecum*) through the consumption of fish and/or cephalopods harbouring these larvae. These parasites have an aquatic life cycle, mainly marine, with marine mammals and piscivorous birds as definitive hosts and crustaceans, cephalopods, and fish as intermediate and/or paratenic hosts [4]. Notably, a case of non-invasive anisakiasis caused by *Hysterothylacium aduncum* [5], a member of the family Raphidascarididae, very close to the anisakids, has also been described. Infection with larvae of the genus *Anisakis* is specifically classified as anisakiosis. However, the term anisakiasis is most commonly used in the medical literature, including instead of anisakidosis, perhaps because the aetiological agent in 97% of cases is the L3 of the complex *Anisakis simplex* sensu lato, specifically the species *A. simplex* sensu stricto (s.s.) and *A. pegreffii* [4]. The symptoms of these infections are mainly gastrointestinal or gastroallergic, and although they can be severe, they usually last no more than two weeks due to the parasite’s death and/or expulsion. On the other hand, numerous cases of human sensitisation to *Anisakis* are known, resulting in mild to severe allergic manifestations after primary infection (allergy to *Anisakis* or allergic anisakiasis). It is not yet excluded that this allergic process can be triggered by other parasites of the same family (genera *Pseudoterranova* and *Contracaecum*) or phylogenetically close (e.g., genus *Hysterothylacium*) since the existence of common antigens among these nematodes has been described [6,7,8,9,10]. Therefore, the consumption of marketed fish carrying these parasites may also pose a potential risk of inducing allergic reactions.

The presence of the larvae of these nematodes in fish has been known since at least the 13th century [11], although Galen (129–216 AD) appears to have been aware of “worms of fishes—interpreted to be encysted nematodes” [12], possibly referring to *Anisakis* larvae encapsulated on the viscera of fish. Linnaeus classified a larval worm from herring, poorly described, as *Gordius marinus* in 1767. Rudolphi described both larvae in fish and adults in porpoises in 1809 and named the species *Ascaris simplex*. In 1845, Dujardin created the subgenus *Anisakis* within the genus *Ascaris*, which included this species. *Anisakis* was finally elevated to a genus by Baylis in 1920 [13]. Although the presence of an *Anisakis*-like larva was described in 1950 from the faeces of an Alaskan Inuit [14], it was not until a few years later (1955–1959) that Dr. Straub, in the Netherlands, established the relationship between “healthy men who suddenly were attacked by violent, abdominal colics with fever” [15] who consumed raw herring and the presence of larvae L3 of *Anisakis simplex* (initially identified as *Eustoma rotundatum*) in their digestive system [15]. This condition was named anisakiasis [16]. This association led the Dutch government to implement prevention and control measures, including freezing, salting, marinating, or smoking herring [11]. These health policies drastically reduced the incidence of human cases, although freezing is now understood as the most effective measure [17].

The expert group of the European Food Safety Authority (EFSA) states, among its main recommendations, that epidemiologic studies should be carried out on fish for human consumption in order to update the knowledge on the risk of human anisakidosis and, therefore, as part of the strategy to be followed for the prevention and control of these infections in humans [18]. In this Special Issue of *Pathogens*, 12 articles have been collected, among which those dedicated to food safety (six papers) predominate. These articles focus on the study of the presence of anisakids in various marine fish of commercial interest, such as *Merluccius* spp., *Conger conger* or *Mullus barbatus*, flatfish such as *Dicologlossa cuneata* and *Citharus linguatula*, and freshwater fish such as the Prussian carp (*Carassius gibelio*) [19,20,21,22,23,24].

However, as an added value to these studies, two of them have addressed the possible negative effects that parasitisation, by *Anisakis* in *Citharus linguatula* and by *Contracaecum* in *Carassius gibelio*, exerts on the development/growth of the host fishes studied both from marine and inland waters [21,24]. The commercial and economic implications in aquaculture and fisheries, in general, are undeniable.

The Special Issue focuses on the presence of anisakids in fish intended for human consumption and the associated risks but also includes two insightful articles on diagnostic strategies for anisakiasis in humans. The first article reports a human case of anisakiasis caused by *A. pegreffii* and proposes the combination of upper gastrointestinal endoscopy with colonoscopy for a better diagnosis and reliable treatment. This allows the elimination of any larvae that may be found in the digestive tract, preventing chronic inflammation and damage caused by the parasite [25] and, at the same time, leading to a faster and safer recovery of the patient. The second article, an observational study, looks at the diagnosis of allergy to *Anisakis* by evaluating the basophil activation test (BAT) [26] based on the use of a novel algorithm previously published by the authors [27]. BAT showed the best diagnostic accuracy (92.45%) and specificity (100%) in detecting *Anisakis*-specific IgE sensitisation in the trial. The authors conclude that although the study has some limitations, it provides a good basis for the future development of clinical guidelines, leading to a better diagnosis of allergy to *Anisakis* [28].

In two other papers, the authors address some aspects of the pathogenesis of *Anisakis* L3, which is poorly understood. In one of them, a human colorectal adenocarcinoma cell line (Caco-2) is used to recreate a model mimicking the inflammatory response that would occur when *Anisakis* larvae invade the human gut. To simulate different situations in the human digestive tract, they exposed the Caco-2 cells to *Anisakis* L3 (the initial contact with the host), to extracellular vesicles released by the larva (*Anisakis*-host communication), and to crude extract (the larval dying). The findings obtained allow the authors to suggest that the parasite could exert an immunomodulatory effect that allows it to survive in the human digestive tract. At the same time, the dying larvae would induce a strong immune response, leading to the expulsion of the parasite, the generation of eosinophilia, and the formation of granulomas [29]. In the second research, the authors carried out a study of the secretome of *A. simplex* s.s. L3, in an attempt to identify excretion–secretion proteins involved in the host–parasite interaction, and subsequent in silico characterisation [30]. They identified 158 proteins, including the allergen known as Ani s 4, as well as 18 potential allergens and nine other proteins potentially involved in the pathogenicity of the parasite. As the authors point out, this appears to be a pathway that may allow a better understanding of the survival and invasion strategies of the *A. simplex* s.s. L3, and perhaps those of other members of the genus *Anisakis,* causing anisakiasis.

Finally, this Special Issue includes two reviews authored by renowned experts in anisakiasis and its aetiological agents. In the first of these, the author discusses the advances in knowledge of the allergic response to *Anisakis* over the last two decades [31]. Although remarkable progress has been made in understanding the pathology caused by *A. simplex*, she believes that significant advances will be made in the coming years, both in the development of rapid diagnostic techniques to better identify patients at risk and in the detection of parasites or their proteins in fish or seafood dishes. She, therefore, warns of the need for health professionals to be aware of the importance and dangers of parasitic zoonoses, especially emerging ones such as anisakiasis, which are largely neglected in many countries.

In the second review, the authors describe the clinical features of anisakiasis and report the main results of the (few) in vivo and in vitro studies carried out to date, highlighting the problem of the lack of standardisation of working methods, which makes it difficult to compare results [32]. As an alternative, the authors propose using three-dimensional organoids, especially for studying pathogenesis, which are still rarely used in parasite studies. Using intestinal organoids for anisakiasis would avoid the fragmentation of available information, as it is a model that better reflects the complexity of the host, in this case, the human host [32]. The authors also propose further studies on extracellular vesicles, as recent studies have enabled their fluorescent labelling in *Anisakis*, facilitating work with them [33], which is very important due to their potential as a mechanism of communication and interaction between parasite and host. Their contents include DNA, proteins, and non-coding RNAs, such as microRNAs (already in evidence in *A. pegreffii* L3 [34]), which play relevant roles in all aspects of cellular life through post-transcriptional gene regulation, potentially promoting the degradation and/or translational repression of target messenger RNAs [35]. Thus, parasites could use their extracellular vesicles to potentially regulate host genes that, among other possible functions, could regulate host immune and inflammatory responses [32]. Indeed, there is already some evidence from work with parasitic nematodes [36,37]. This opens up a very interesting and promising future in the study and understanding of host–parasite interactions, potentially leading to better, perhaps targeted, therapies against parasites.

In summary, this Special Issue provides the interested reader with a series of interesting articles on different aspects of anisakiasis, giving an insight into the current situation and the bright future that can be envisaged depending on the application of the new techniques that are being developed.
